# The role of IL-6 and osteoprotegerin in bone metabolism in patients with Graves’ disease

**DOI:** 10.3906/sag-2105-1

**Published:** 2021-11-11

**Authors:** Tülay OMMA, Çiğdem YÜCEL, Erdim SERTOĞLU, Sevde Nur FIRAT, Cavit ÇULHA, Taner ÖZGÜRTAŞ

**Affiliations:** 1 Department of Endocrinology and Metabolism, Ankara Training and Research Hospital, University of Health Sciences, Ankara, Turkey; 2 Department of Clinical Biochemistry, Ankara Numune Training and Research Hospital, University of Health Sciences, Ankara, Turkey; 3 Department of Clinical Biochemistry, Ankara Gülhane Training and Research Hospital, University of Health Sciences, Ankara, Turkey

**Keywords:** Graves’ disease, osteoprotegerin, receptor activator of NF-kB ligand, interleukin-6, bone, immunity

## Abstract

**Background/aim:**

Increased bone turnover is a hallmark of hyperthyroidism. The underlying factors of how thyroid hormones affect bone cells are still under the spotlight. Previous studies indicated serum osteoprotegerin (OPG), receptor activator of NF-kB ligand (RANKL), and interleukin-6 (IL-6) as mediators of the effect of thyroid hormones on bone metabolism. Ultimately, the present research aimed to examine the association of IL-6 with OPG and RANKL in patients with hyperthyroidism.

**Materials and methods:**

We carried out this study with 39 newly diagnosed and untreated Graves’ patients and 43 healthy controls. In addition to routine tests, we measured serum OPG, RANKL, and IL-6 levels.

**Results:**

Mean age and sex distribution were similar in both groups. The hyperthyroid group had significantly higher OPG (p = 0.002) and IL-6 (p < 0.001) levels, but RANKL levels were significantly lower in this group (p < 0.001). We found OPG not to correlate with free T4 and T3, while it had a moderate and negative correlation with thyrotropin (TSH) (r = −0.372, p = 0.001). IL-6 had no correlation with OPG but positively correlated with free T4 (r = 0.445, p < 0.001) and free T3 (r = 0.326, p = 0.035). It also negatively correlated with RANKL (r = −0.247, p = 0.033).

**Conclusion:**

Maintaining skeletal development and integrity is partially regulated by a normal balance of thyroid hormones. We concluded that increases in serum OPG and IL-6 levels accompanied hyperthyroidism. However, excessive levels of the hormones might cause drops in serum RANKL levels. Our results suggested that OPG, RANKL, and IL-6 might be involved in the cross-talking among immunity, thyroid function, and bone metabolism in the case of hyperthyroidism.

## 1. Introduction

Graves’ disease (GD), a common autoimmune disorder, is characterized by thyrotoxicosis, diffuse goiter, and infiltrative ophthalmopathy [1]. The etiology and pathogenesis of GD are not fully understood, but the role of intrathyroidal thyrotropin receptor antibodies (TRAb) is well known [2]. As TRAb is produced by immunological mechanism, intrathyroidal inflammatory cells can secrete proinflammatory cytokines to support local inflammation and autoimmune process [3]. Interleukin-6 (IL-6) is a significant pleiotropic proinflammatory cytokine produced in various tissues such as bone, thyroid, and blood mononuclear cells [4, 5]. These mononuclear cells also express thyroid hormone receptors [6]. Lakatos et al. showed that serum concentrations of IL-6 produced by blood mononuclear cells in patients with toxic nodular goiter or GD were significantly higher than in controls, suggesting that IL-6 is involved in the pathogenesis of GD [7]. In addition, Lv et al. revealed that IL-6 expression was higher in thyroid tissue in elderly hyperthyroid patients compared to the control group [8]. Although several genetic studies have evaluated the association between the IL-6 -174 G/C polymorphism and individuals’ susceptibility to GD, no consensus has been reached so far, possibly due to study population differences and limited sample sizes [9, 10].

Elevated thyroid hormones directly stimulate bone cells, resulting in increased bone turnover [11]. Although hyperthyroidism causes an increase in both bone formation and resorption, the underlying mechanisms are still under scrutiny. It was previously shown that the effects of thyroid hormones on bone are mediated by osteotropic cytokines such as IL-6 and IL-8 [12]. Osteoprotegerin (OPG) and its cognate ligand, receptor activator of NF-kB ligand (RANKL), mediate paracrine signaling between osteoblasts and osteoclasts [13]. In general, upregulation of RANKL is associated with downregulation of OPG, and an increase in the RANKL/OPG ratio favors osteoclastogenesis. OPG is predominantly produced by osteoblasts and inhibits RANKL-RANK interaction, leading to inhibition of osteoclast development and osteoclastic bone resorption [14]. OPG-deficient mice show intense osteoporosis caused by enhanced adult stage osteoclastogenesis [14]. Because patients with cardiovascular and autoimmune diseases have been reported to have elevated serum OPG levels, it is likely to imply a possible relationship between bone metabolism, vascular biology, and immune function [15]. RANKL is considered to have the ability to mature osteoclasts when macrophage colony stimulating factor (M-CSF) is not present. Bone and lymphoid tissues are where RANKL mRNA is mainly expressed. Eventually, disruption of the balance of RANKL and OPG can lead to undesirable bone resorption [16]. This system is also affected by factors such as cytokines (including TNF-alpha, IL-1), glucocorticoids, parathyroid hormone (PTH), estrogen, and vitamin D [17].

The literature trying to correlate thyroid hormones and bone metabolism indicates that tri-iodothyronine (T3) stimulates osteoblast proliferation by increasing the expression of osteocalcin, alkaline phosphatase (ALP), type 1 collagen and insulin-like growth factor-I [18]. Several other studies have examined the effect of thyroid hormones on serum IL-6 and its relationship to bone loss [19]. IL-6 can exert its inhibitory effect on bone formation in two ways: a direct effect through gp130-STAT 1/3 signaling or an indirect effect by manipulating the balance between OPG and RANK and its ligand (RANKL) [20].

In vitro studies on organ cultures of fetal rat limb and neonatal mouse calvariae also showed an unmediated stimulation of bone resorption by T3 [21]. Miura et al. revealed that mRNA expression of RANKL in bone was enhanced by T3 [22]. Nonetheless, some other studies found how T3 stimulates osteoclastogenesis was divergent when compared to RANKL. Kanatanı et al. concluded that OPG did not inhibit T3-induced osteoclast-like cell development from osteoclast precursor cells, while it completely inhibited their formation, induced by RANKL and M-CSF [23]. Suda et al. discovered T3 not to influence IL-6 concentrations and prostaglandin E2, known to upregulate RANKL expression in osteoblast/stromal cells. This result supports that the primary regulator in T3-induced osteoclast differentiation may not be the RANKL/OPG system [24]. Based on these contradictory results, the present study aimed to investigate the relationship between IL-6, which is a part of immunity, and OPG and RANKL, which are involved in bone metabolism in patients with hyperthyroidism.

## 2. Materials and methods

### 2.1. Study protocol

It was a cross-sectional study and included newly diagnosed and untreated GD patients (n = 39) and euthyroid healthy subjects with no thyroiditis or any other acute or chronic diseases (n = 43). The participants were aged between 18–65 and selected among those visiting the endocrinology outpatient clinics of Ankara Training and Research Hospital, Turkey.

The diagnosis of Graves’ disease was confirmed by the presence conventional symptoms of hyperthyroidism, biochemically overt hyperthyroidism, enlarged thyroid gland and increased blood supply on ultrasound, anti-thyroid peroxidase antibody (anti-TPO) or anti-thyroglobulin antibody (anti-Tg) positivity, TRAb positivity in all patients, and increased radioactive iodine uptake in thyroid scintigraphy. Some patients also had ophthalmopathies at the time of diagnosis.

The inclusion criteria were as follows: (i) no acute or chronic diseases (recent infection/inflammation, chronic obstructive pulmonary disease, autoimmune diseases, malignancy, chronic renal or liver failure, and heart failure) besides hyperthyroidism, (ii) no medicine treatment affecting either the immune system or bone metabolism within a year before the enrollment, (iii) no pregnancy within the last 18 months before enrolment; (iv) no medical treatment for hyperthyroidism, (v) no risk factors for an altered bone turnover (connective tissue disorders, immobilization, malabsorption, history of bone fracture within the last 12 months), (vi) no history of thyroid operation or prior exposure to radioiodine or external radiation.

We measured some parameters of the participants’ blood samples: serum levels of free-thyroxine (free T4), free T3, thyrotropin (TSH), TRAb (except control group), anti-Tg, calcium (Ca), anti-TPO, magnesium (Mg), phosphorus (P), PTH, serum 25-hydroxyvitamin D (25(OH)D), OPG, RANKL, and IL-6.

We obtained venous blood samples following overnight fasting from the antecubital vein between 9:00 and 11:00 a.m. at a resting position. We centrifuged the samples at 1500 g for 10 min. We aliquoted separated sera were into Eppendorf tubes and stored them at −80 °C till the analyses. As a part of their clinical evaluation, we sought demographic and clinical data from the subjects. We matched the patients with the healthy controls by age and sex. We performed radioactive iodine uptakes, thyroid ultrasonography, and scintigraphy to diagnose GD.

Ankara Numune Training and Research Hospital Ethical Committee granted the relevant approval for our study protocol (Number: 2300/2018). We obtained written informed consent from each participant and carried out the research following the principles of the Declaration of Helsinki.

### 2.2. Laboratory analysis

We measured serum levels of free T3, free T4, TSH, anti-Tg, and anti-TPO using Beckman Coluter Inc. (CA, USA) DxI 800 Analyzer with a two-site immunoenzymatic assay, according to the manufacturer’s instructions. Serum TRAb was tested using Siemens Healthcare GmbH (Erlangen, Germany) Immulite 2000 immunoassay analyzer following the automated sandwich chemiluminescent immunoassay method. We detected serum IL-6 levels with Beckman Coulter Access 2 autoanalyzer with simultaneous one-step immunoenzymatic assay.

We determined serum OPG and RANKL levels with commercial human ELISA (quantitative double antibody sandwich ELISA method) test Kit (My Biosource Inc. CA, USA) according to the manufacturer’s protocol. For OPG, the detection range of the kit was between 62.5 and 4000 pg/mL, and the assay sensitivity was 2 pg/mL. For RANKL, the measurement range of the kit was between 15–240 pmol/L, and the assay sensitivity was 1 pmol/L. For both parameters intraassay precision was <8% and inter-assay precision was <12%.

### 2.3. Statistical analysis

We run all statistical analyses of the data on the Statistical Package for Social Sciences (SPSS) program version 21.0 for Windows (SPSS Inc., Chicago, IL). The Kolmogorov–Smirnov test was performed to check the normality of distribution of the data. We showed normally distributed continuous and categorical values as mean±SD and numbers and percentages, respectively. Using the Chi-Square test, we explored the differences between the groups by the categorical variables. We expressed non-normally distributed parameters as medians with interquartile range [IQR]. We compared the groups by continuous variables utilizing the Student’s t-test or Mann–Whitney U test. We calculated Pearson’s or Spearman’s rank correlation coefficient to examine the correlations of the study variables. We considered differences to be statistically significant when p < 0.05. The trial had over 94% power for OPG and RANKL calculated with the G*Power program [25].

## 3. Results

Thirty-nine newly diagnosed graves patients (28 female, 11 male) and forty-three healthy controls (33 female, 10 male) participated in the study. Mean age and sex distribution were similar in both groups. Table 1 presents the baseline characteristics of the participants. Although the groups did not differ by 25(OH)D (p = 0.45), Ca (p = 0.80), and Mg (p = 0.73), there were significant differences between them by PTH, P, and ALP levels (p < 0.001). PTH and P levels were lower in GD patients. The hyperthyroid patients had significantly lower TSH (p < 0.001) and higher free T4 and free T3 concentrations than control cases (p < 0.001 for free T4; p < 0.001 for free T3). We measured anti-TPO and anti-Tg autoantibodies for both groups, but TRAb was only measured in the patient group and the median of TRAb was 29.9 (IQR = 56.1). The groups also significantly differed by the anti-TPO and anti-Tg levels (p < 0.001 for anti-TPO and p = 0.03 for anti-Tg).

OPG (p = 0.002) and IL-6 (p < 0.001) were significantly higher in the patient group, which had significantly lower RANKL (p < 0.001). OPG showed a weak and positive correlation with ALP (r = 0.264, p = 0.018) but a negative one with TSH (r = −0.372, p = 0.001). We found no correlation between OPG and other study parameters. There were positive correlations between RANKL levels and TSH (r = 0.493, p < 0.001), PTH (r = 0.291, p = 0.01), P (r = 0.508, p < 0.001), but negative correlations between RANKL and IL-6 (r = −0.247, p = 0.03), free T3 (r = −0.386, p = 0.01), free T4 (r = −0.485, p < 0.001), anti-TPO (r = −0.343, p = 0.003). IL-6 had no correlation with OPG but positively correlated with free T4 (r = 0.445, p < 0.001), free T3 (r = 0.326, p = 0.035), anti-TPO (r = 0.337, p = 0.007), and ALP (r = 0.426, p < 0.001). It also negatively correlated with RANKL (r = −0.247, p = 0.033), P (r = −0.347, p = 0.002), PTH (r = 0.322, p = 0.006), and TSH (r = −0.488, p < 0.001). Table 2 demonstrates the results of the correlation analyses with all participants. When correlation analysis was performed only with the results of graves patients, OPG was not correlated with any test. RANKL was only negatively correlated with free T4 (r = −0.334, p = 0.038), and IL-6 was only positively correlated with ALP (r = 0.441, p = 0.06).

## 4. Discussion

As a result, we discovered hyperthyroid patients had elevated serum OPG and IL-6 levels and lowered RANKL concentrations compared to the healthy controls. These increases were associated with the entity of thyroid dysfunction and an increase in bone turnover. We found OPG not to correlate with free T4 and T3, while it had a moderate and negative correlation with TSH. IL-6 had no correlation with OPG but positively correlated with free T4 and free T3. It also negatively correlated with TSH and RANKL. Also RANKL was positively correlated with TSH but negatively correlated with free thyoid hormones.

It is well-known that the increase in thyroid hormones affects bone structure and strength and eventually causes bone loss. Nevertheless, the underlying mechanism of how thyroid hormone functions on bone loss is still undercover. The bone cycle falls to nearly half of its standard turnover time (3–4 months) in the case of hyperthyroidism. Such an accelerated rate of turnover generates an increased number of osteoclast resorption sites and ultimately boosts bone resorption ratio, causing osteoporosis and fragility fracture.

Previously, few studies attempted to examine OPG in thyroid dysfunction, and these studies reported inconsistent results. Nagasaki et al. showed that serum OPG levels in hypothyroid patients were significantly higher than normal controls [26]. It has, therefore, been suggested that by acting as an inhibitor of osteoclastogenesis, OPG may establish a link between hypothyroidism and a reduction in bone resorption. OPG levels in overt thyrotoxicosis were evaluated in several trials; however, the mechanism of how thyroid hormone dysfunctions modify serum OPG has not exactly yet been revealed. Besides, thyrotoxicosis, which is known to be associated with osteoclastogenesis, is predicted to be associated with decreased OPG levels. However, Amato et al. showed that serum OPG concentrations were higher in hyperthyroid patients than in euthyroid subjects [27]. Mochizuki et al. demonstrated that taking anti-thyroid drugs significantly reduced serum OPG levels [28]. Similarly, we observed that serum OPG concentrations were increased in hyperthyroid patients compared to euthyroid subjects, regardless of age. This finding was also consistent with other data suggesting that thyroid hormones have physiological effects on OPG production. Varga et al. revealed that thyroid hormone promoted OPG mRNA levels in mature MC3T3-E1 osteoblastic cells but not in bone marrow stromal ST2 cells or preosteoblastic MC3T3-E1 cells [29]. In addition, previous studies have shown that OPG and RANKL are produced by thyroid follicular cells and OPG mRNA is regulated by TSH and cytokines [30]. Hofbauer et al. determined that OPG mRNA levels were three times higher in thyroid surgery specimens from Graves’ patients, whereas Lv et al. demonstrated increased IL-6 expression in hyperthyroid tissue, which indicates the possible effect of autoimmunity [8, 30]. Weetmann et al. found that IL-6 increased intrathyroidally in GD, but not in serum [4]. In this case, our study confirms that serum IL-6 is not only increased by blood mononuclear cells and bone in hyperthyroid patients but also produced by the thyroid tissue in association with OPG. This proposition suggests that the increase in OPG in both hypothyroid and hyperthyroid patients may be due to the significant effect of IL-6 and autoimmunity.

Our statistical analyses revealed that serum OPG levels did not correlate with free T3 or free T4. Nevertheless, Mochizuk et al. found it to correlate with free T4 but not with free T3, while Amato et al. showed a correlation of OPG levels with free T3 but not with free T4 [27, 28]. Our findings also revealed that OPG levels negatively correlated with TSH levels, as opposed to the conclusions from Özdemir et al., suggesting that thyroid hormone status might affect OPG [31]. Besides, TRAb is known to be used to diagnose GD and interacts with and stimulates the TSH receptor expressed in specific tissues. However, we did not establish any correlation between OPG and TRAb.

Since some studies have shown that TSH has inhibitory effects on skeletal remodeling independent of thyroid hormone, it can be argued that TRAb or TSAb have an inhibitory role in bone metabolism [32]. These observations are consistent with the hypothesis that bone metabolism is regulated by TSH through different mechanisms independent of the RANKL-OPG pathways [30]. Abe et al. showed that TSH has a critical role in maintaining bone mass independent of thyroid hormones. In their study, osteoporosis was increased in TSH receptor (−/−) knockout mice. They speculated that the skeletal loss that occurs in hyperthyroidism is due to low TSH levels, as opposed to simply increased thyroid hormones [33].

It is known that thyrotropin receptor (TSHR) is expressed in osteoclasts and osteoblasts [34]. In one study, intermittent low-dose TSH treatment was shown to prevent ovariectomy-induced bone loss in mice. In another study, OPG transcription was significantly increased in response to TSH [34]. TSH can also trigger the expression of type 2 iodothyronine deiodinase in osteoblasts. Therefore, as in hyperthyroidism, suppressed TSH levels can cause bone loss. However, the main view on this subject is that TSH is a direct negative regulator of bone turnover. Although the effects of agonist TRAb on the skeleton are still unknown, it is more likely that these antibodies bind to and mobilize the skeletal TSHR. There is a study that supports this concept, showing an inverse association of bone density loss with TRAb levels.

IL-6 is one of the major proinflammatory cytokines whose serum levels are known to be elevated in GD [35]. IL-6 can enhance the development and production of thyroid receptor antibodies during the GD course [36]. In this context, Grubeck-Loebenstein et al. showed that there are follicular cells synthesizing IL-6 in the thyroid glands of GD patients [37]. Although evidence to date indicates that IL-6 is critical in the early phase of osteoclast differentiation, IL-6 has not been found to mediate the effect of T3 on osteoclast differentiation [37]. In addition, it was previously revealed that IL-6 reinforced RANKL mRNA expression and hindered bone formation [20]. In our study, the hyperthyroid group had significantly higher IL-6 levels than the controls.

Although the literature shows conflicting results on IL-6 levels regarding thyroid status, we found that IL-6 was strongly and negatively correlated with TSH but positively correlated with free T3 and free T4. IL-6 correlated positively with anti-TPO and ALP, while negatively correlated with RANKL, P and PTH. Although IL-6 is secreted from normal parathyroid tissue and with PTH in hyperparathyroidism, the negative correlation of IL-6 with PTH in our study can be explained by slight increases in Ca levels [38].

In our study, an increase in favor of hyperthyroidism was found in ALP. Although the groups did not differ in terms of Ca, Mg and 25(OH)D, PTH was lower in hyperthyroid patients. In vitro, PTH is known to inhibit OPG mRNA expression in cultured bone marrow [39]. Serum OPG levels also have a negative relationship with PTH in healthy men over 40 years. Increased serum Ca caused by increased mobilization of bone minerals in hyperthyroid patients suppresses serum PTH levels. The multiple regression analysis by Mochizuki et al. resulted in OPG having an independent, significant, and negative correlation with PTH, but it was not the case with Ca and P, which indicates that PTH is a remarkable predictor of OPG during thyrotoxicosis treatment [28]. Nonetheless, we could not reach any correlation between OPG and PTH, Ca, P, and 25(OH)D. Although we did not measure it in our study, 1,25-dihydroxyvitamin D (1,25-(OH)_2_D) inhibits T3-induced expression of OPG mRNA, but low levels of 1,25-(OH)_2_D in hyperthyroidism are likely to explain the high OPG levels in GD patients [27, 29]. We can say that elevated serum OPG levels mean a stabilizing mechanism against higher bone resorption caused by hyperthyroidism

On the other hand, there is also evidence that there is no relationship between thyroid status and OPG and RANKL levels. Giusti et al. showed that patients with different thyroid hormone levels and levothyroxine doses had similar OPG and RANKL levels [40]. In another study, it was demonstrated that recombinant TSH did not affect OPG and RANKL levels in differentiated thyroid carcinoma [41]. It has been shown that T3 has a direct effect on the proliferation and differentiation of human osteoblast-like cells, and T3 is unable to stimulate osteoclastic bone resorption in the absence of osteoblasts [42]. Kanatani et al. revealed that although T3 increased OPG mRNA expression in bone cells, it had no effect on RANKL mRNA expression [23]. These results imply that the RANKL/OPG system is not a major regulator of T3-induced osteoclast differentiation.

The present study had a strength, as well as some limitations. Its primary strength was related to the homogeneity of the patient groups included. Regarding the limitations, we designed the study to be cross-sectional with no follow-up data and post-treatment results of the study groups. Also, the sample size was relatively small.

In conclusion, we found that the regular balance of thyroid hormones could regulate skeletal development and help the preservation of skeletal integrity. Our study showed that hyperthyroidism was associated with an elevation in serum OPG and IL-6 levels while a decrease in serum RANKL in relation to the excess in thyroid hormones. The results revealed that IL-6 had an important function in the stimulation of the RANKL-RANK/OPG mechanism, which was strongly enhanced when the presence of accelerated bone turnover such as thyrotoxicosis. Finally, OPG, RANKL, and IL-6 might be involved in the cross-talking among immunity, thyroid function, and bone metabolism in the case of hyperthyroidism. Further and more studies will help elucidate this proposition.

## Figures and Tables

**Table 1:** Baseline characteristics of the participants.

	Graves patients (n = 39)	Controls (n = 43)	p value
**Age (years)a**	40.03 ± 11.28	37.84 ± 7.31	0.29
**TSH (mIU/L)a**	0.02 ± 0.03	1.95 ± 1.14	<0.01
**Free T4 (ng/dL)a**	4.33 ± 2.07	0.98 ± 0.21	<0.01
**Free T3 (ng/L)a**	16.14 ± 8.55	3.36 ± 0.41	<0.01
**Anti-TPO (IU/mL)b**	81.1 (259.5)	3.65 (15.72)	<0.01
**ALT (U/L)a**	30.23 ± 17.46	20.53 ± 14.22	<0.01
**AST (U/L)a**	24.38 ± 12.13	20.09 ± 5.85	0.07
**ALP (U/L)b**	83.0 (65.5)	58.0 (29.0)	<0.01
**GGT (U/L)b**	29.0 (26.0)	13.0 (13.0)	<0.01
**Ca (mg/dL)a**	9.39 ± 0.54	9.37 ± 0.43	0.80
**P (mg/dL)b**	1.80 (0.25)	3.16 (1.41)	<0.01
**Mg (mmol/L)a**	4.07 ± 1.19	3.99 ± 0.59	0,73
**PTH (pg/mL)b**	23.3 (16.75)	50.4 (25.4)	<0.01
**25(OH)D (μg/L)b**	11.28 (7.49)	10.35 (9.80)	0.45
**OPG(pg/mL)a**	887.07 ± 342.61	658.26 ± 301.01	<0.01
**RANKL (pmol/L)a**	34.09 ± 11.57	54.07 ± 18.35	<0.01
**IL-6 (pg/mL)b**	3.58 (6.19)	1.44 (1.18)	<0.01
**RANKL/OPGb**	0.04 (0.03)	0.08 (0.11)	<0.01

a: Mean ± standard deviation.

b: Median (interquartile range).

p: val<0.05 denoted as statistically significant (in bold).

* Student’s t-test; Mann–Whitney U test.

**Abbreviations:**TSH, Thyrotropin; free T4, free thyroxine; free T3, free triiodothyronine; Anti-TPO, Anti-thyroid peroxidase antibody; ALT, alanine aminotransferase; AST, aspartate aminotransferase; ALP, alkaline phosphatase; GGT, gamma-glutamyl transferase; Ca, calcium; P, phosphorus; Mg, magnesium; PTH, parathormone; 25(OH)D, 25 hydroxy vitamin D; OPG, osteoprotegerin; RANKL, receptor activator of NF-kB ligand; IL-6, serum interleukin 6; RANKL/OPG, RANKL/OPG ratio; TRAb, thyrotropin receptor antibodies.

**Table 2:** Correlation table for all participants.

Parameters	OPG		RANKL		IL-6	
	r value	p value	r value	p value	r value	p value
**Age (years)**	−0.095	0.397	−0.107	0.339	0.226	0.052
**Ca (mg/dL)**	0.097	0.387	0.100	0.373	−0.001	0.996
**P (mg/dL)**	−0.197	0.077	.508**	**<0.001**	−.347**	**0.002**
**Mg (mmol/L)**	0.130	0.258	−0.018	0.876	−0.131	0.267
**ALP (U/L)**	.264*	**0.018**	−0.099	0.383	.426**	**<0.001**
**PTH (pg/mL)**	−0.135	0.239	.291**	**0.010**	−.322**	**0.006**
**25(OH)D (μg/L)**	0.033	0.769	−0.104	0.356	0.040	0.735
**TSH (μIU/mL)**	−.372**	**0.001**	.493**	**<0.001**	−.488**	**<0.001**
**Free T3 (ng/L)**	−0.037	0.813	−.386**	**0.010**	.326*	**0.035**
**Free T4 (ng/dL)**	0.209	0.060	−.485**	**<0.001**	.445**	**<0.001**
**Anti-TPO (IU/mL)**	0.199	0.097	−.343**	**0.003**	.337**	**0.007**
**Anti-Tg(IU/mL)**	−0.093	0.614	−0.182	0.319	0.151	0.419
**TRAb (U/L)**	−0.035	0.834	0.218	0.189	0.024	0.887
**OPG (pg/ml)**	1		−0.162	0.146	0.221	0.056
**RANKL (pmol/l)**	−0.162	0.146	1		−.247*	**0.033**
**IL-6 (pg/ml)**	0.221	0.056	−.247*	**0.033**	1	
**RANKL/OPG**	−.781**	**<0.001**	.684**	**<0.001**	−.363**	**0.001**

** Significant at the 0.01 level (2-tailed).

* Significant at the 0.05 level (2-tailed).

r: Correlation coefficient; Pearson’s correlation coefficient (normal distribution) and Spearman’s rank coefficient (not normal distribution).

**Abbreviations:** Ca, calcium; P, phosphorus; Mg, magnesium; ALP, alkaline phosphatase PTH, parathormone; 25(OH)D, 25 hydroxy vitamin D; TSH, thyrotropin; free T3, free triiodothyronine; free T4, free thyroxine; Anti-TPO, Anti-thyroid peroxidase antibody; Anti-Tg, Anti-thyroglobulin antibody; TRAb, thyrotropin receptor antibodies;OPG, osteoprotegerin; RANKL, receptor activator of NF-kB ligand; IL-6, serum interleukin 6.
